# Successful multi-modality approach in management of human leukocyte antigen-B27 associated fulminant panuveitis - rare presentation

**Published:** 2018

**Authors:** Ashok Kumar, Vikas Ambiya, Gaurav Kapoor, Amit Arora

**Affiliations:** * Army College of Medical Sciences & Base Hospital, Delhi Cantonment, New Delhi, India -110010

**Keywords:** HLA-B27, posterior uveitis, Ozurdex, papillitis, pars plana vitrectomy, immunosuppressive agents

## Abstract

**Objective:** To report a multimodality approach in the management of human leukocyte antigen B27 (HLA-B27) associated fulminant posterior uveitis, an uncommon presentation, with good visual and anatomical recovery.

**Methods:** A 33-year-old young male presented with HLA-B27 associated severe posterior uveitis, which is a relatively uncommon presentation. The patient had severe vitritis with papillitis, which was sequentially and stepwise managed with oral steroids, pars plana vitrectomy, immunosuppressive agents and sustained release intravitreal steroid implant.

**Results:** The patient had a good recovery of vision with complete resolution of inflammation and without any long-term complication.

**Conclusion:** HLAB27 positivity can be associated with an uncommon presentation of fulminant posterior uveitis that requires a judicious and stepwise multimodality approach in its management, and can have a good visual and anatomical outcome as demonstrated in our case.

## Introduction

Human leukocyte antigen B27 (HLA-B 27) associated uveitis is characterized by recurrent alternating acute unilateral inflammation in the anterior chamber. It is the second commonest cause of anterior uveitis, following idiopathic uveitis [**1**]. Posterior segment involvement in HLA-B27 associated uveitis is relatively uncommon but has been shown to occur in up to 17% of the patients with HLA-B27 associated uveitis. This may take the form of posterior vitritis, vasculitis, or papillitis [**2**].

The treatment of noninfectious posterior uveitis remains a challenge and the initial management is predominantly with systemic corticosteroids. It has been proposed that up to two thirds of the patients with posterior uveitis can be treated with corticosteroids alone. However, long-term systemic steroids are known to cause serious adverse effects in many patients, and immune modulatory treatment is commonly suggested as steroid-sparing approach. While immune modulatory agents can be very effective, they may have serious and potentially life-threatening adverse effects [**3**,**4**]. Some of these patients with relentless uveitis require surgical treatment in form of pars plana vitrectomy, which both helps in the reduction of inflammatory load as well as provides sample for laboratory investigations.

We report a case of HLA-B27 associating very severe posterior uveitis mimicking endogenous endophthalmitis, a relatively uncommon presentation, successfully managed using sequential multi-modality approach of oral immunosuppression, surgical approach, and intravitreal biodegradable steroid implant, with a good visual and functional recovery.

## Case report

A 32-year-old healthy Indian male presented with complaints of painful diminution of vision in his left eye for the past three days. The ocular evaluation revealed a best corrected visual acuity (BCVA) of 20/ 20 in the right eye and 20/ 80 in the left eye, with severe anterior chamber inflammation and hypopyon. There was a mild spillover inflammation seen in the vitreous cavity but third order vessels in the fundus could be visualized.

**Fig. 1:**
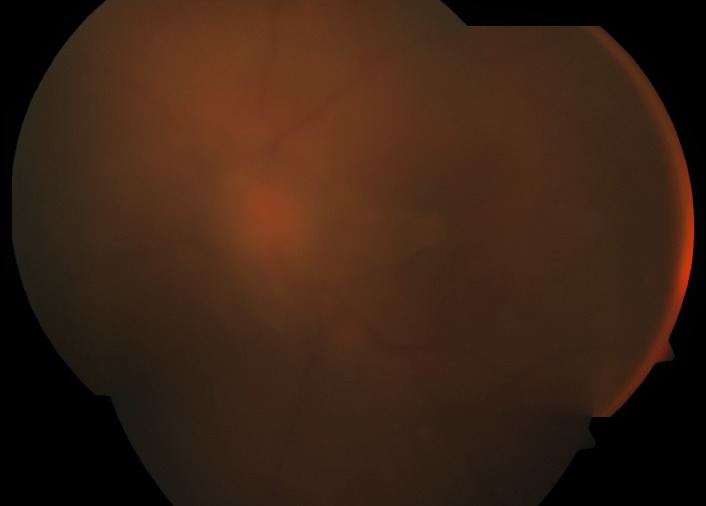
Color fundus photograph of left eye showing intense vitritis, with optic disc and vessels faintly visible three days after starting therapy with oral steroids

The patient was started on intense topical steroids and cycloplegics. All routine systemic investigations including HLA-B27, and X-ray sacroiliac joint were done. He was found to be HLA-B27 positive by deoxyribonucleic acid based molecular method: single specific primer- polymerase chain reaction (PCR). Over the next three days, the anterior segment inflammation considerably reduced, with the resolution of the hypopyon, but the patient noticed profound diminution of vision in the same eye, dropping to 20/ 400. The examination of the left eye revealed intense vitritis with media clarity decreased to Grade III, with the optic disc and vessels being faintly visible (**Fig. 1**), and optical coherence tomograph (OCT) showing macular fold and gross macular thickening. He was started on a high dose of oral steroids (1.5mg/ Kg body weight) in order to control the inflammation over the next one week, but the posterior segment inflammation persisted. In view of hypopyon at presentation and dense vitritis with no response to high dose oral steroids, a differential diagnosis of endogenous endophthalmitis was kept in mind and the patient was subjected to a diagnostic and therapeutic pars plana vitrectomy. The vitreous sample was negative for bacteria and fungi on smear as well as culture. The vitreous sample was negative for Mycobacterium tuberculosis (MTB) by PCR (Xpert MTB RIF assay G4).

**Fig. 2:**
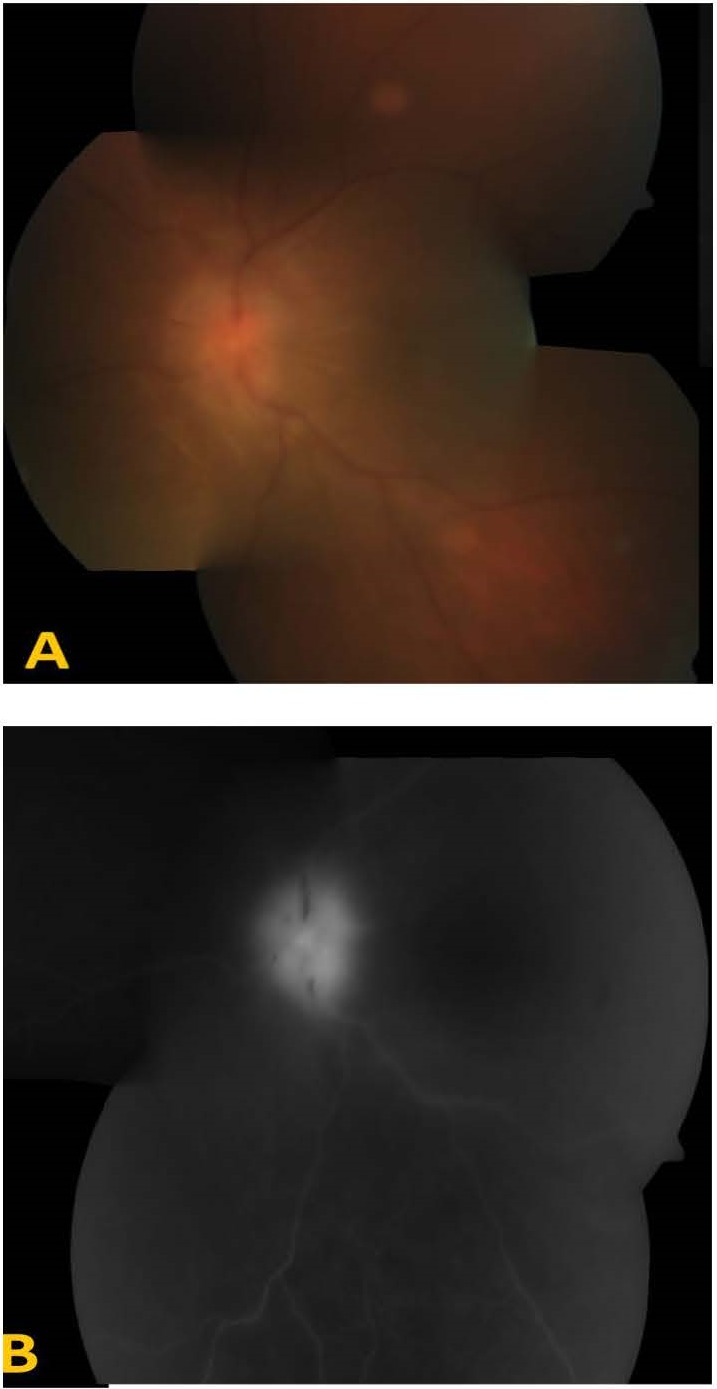
A: Color fundus photograph of left eye showing optic disc edema and macular folds. B: Fundus fluorescein angiogram of the same eye showing leakage from the optic disc

There was a slight improvement in the posterior segment inflammation as well as media clarity after the surgical procedure, with examination revealing optic disc edema, as well as macular folds on fundus photograph (**Fig. 2**) as well as on OCT. When the patient started showing adverse effects of high dose steroid therapy, he was started on oral Methotrexate 15 mg/ weekly, with the posterior segment inflammation and disc edema still persisting, and with a mild improvement in vision to 20/ 200. The patient developed an idiosyncratic reaction to methotrexate in the form of raised serum transaminases by the third week of methotrexate therapy, which was therefore stopped. 

**Fig. 3:**
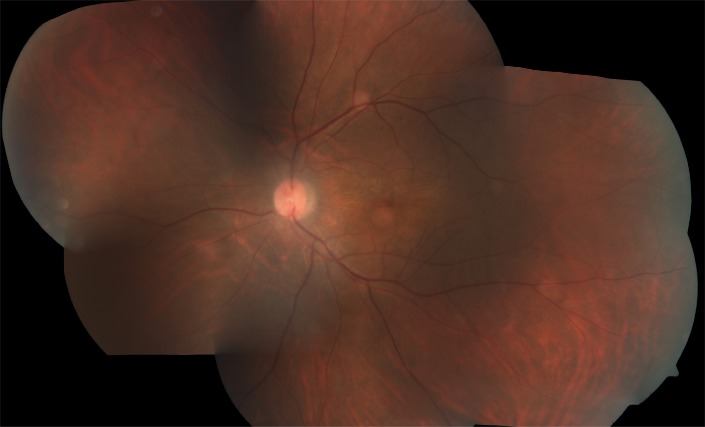
Color fundus photograph of left eye showing complete resolution of dense vitritis and disc edema with visual acuity improving to 20/ 40 four weeks after the injection of intravitreal dexamethasone implant (Ozurdex)

The patient continued to have a significant posterior uveitis with optic disc edema despite being on high dose oral steroids, having undergone pars plana vitrectomy, and having been on immunosuppressive therapy. In view of the long-term requirement of an anti-inflammatory agent in this case, a sustained release dexamethasone implant (Ozurdex) was placed in the vitreous cavity with slow tapering of oral steroids. The patient showed a good response to the implant with a mild increase in intraocular pressure, which was controlled with topical anti-glaucoma medications. There was a complete resolution of the dense vitritis and disc edema with visual acuity improving to 20/ 40 four weeks after Ozurdex implant injection (**Fig. 3**).

## Discussion

Steroids administered either by topical, intraocular or parenteral route remain the gold standard therapy for a large proportion of cases of uveitis and further immunosuppression is usually reserved for selective cases. In addition to steroids, anti-VEGF agents, methotrexate, anti-TNF-α, biologicals and sirolimus are the different local treatment options available to the physician [**5**,**6**]. There is no definitive algorithm available/ practiced for the use of these drugs in case of severe posterior uveitis but steroids are definitively the first line of treatment.

Our case was unique in presentation, with a typical HLA-B27 associated anterior uveitis in the beginning, which later progressed to a fulminant posterior uveitis that mimicked endogenous endophthalmitis. Posterior segment involvement in HLA-B27 associated uveitis is uncommon with an incidence of less than 17% [**2**]. Our case was initially managed with a high dose of oral steroids with no response. He was then taken up for surgical management in view of fulminant posterior segment inflammation, which helped us rule out infective etiology and also provided vitreous sampling for tissue diagnosis to rule out masquerade syndrome. Sanghavi et al. described a series of cases with fulminant posterior uveitis, but not all required management with surgery and high dose steroids or with immunosuppressive agents [**7**]. Castillo et al. described another case of HLA-B27 associated diffuse vitritis but was adequately managed with topical and oral steroids only [**8**].

Our patient did not respond well to oral steroids and to surgery, and was given oral methotrexate, which was also stopped after three weeks due to adverse effects. In view of persistent posterior segment inflammation, and long term side effects of high dose systemic steroid, he was managed with sustained release intravitreal dexamethasone implant and tapering dose of oral steroids. The patient showed a good response to the steroid implant with a complete resolution of vitreous inflammation and papillitis and a good visual recovery. 

This case highlighted a stepwise multimodality approach in the management of an uncommon and a fulminant presentation of HLA-B27 associated uveitis, with a good visual and anatomical recovery.

**Acknowledgements**

Nil.

**Source of funding**

Nil.

**Disclosure**

Nil.
